# Diagnostic Value of Diffusion-weighted Magnetic Resonance (MR) Imaging, MR Perfusion, and MR Spectroscopy in Addition to Conventional MR Imaging in Intracranial Space-occupying Lesions

**DOI:** 10.7759/cureus.6409

**Published:** 2019-12-18

**Authors:** Zeynep B Aydın, Hasan Aydın, Erdem Birgi, Baki Hekimoğlu

**Affiliations:** 1 Radiology, Hitit University Erol Olcok Training and Research Hospital, Çorum, TUR; 2 Radiology, Dr. Abdurrahman Yurtaslan Oncology Training and Research Hospital, Ankara, TUR; 3 Radiology, Diskapi Yildirim Beyazit Training and Research Hospital, Ankara, TUR

**Keywords:** brain tumor, mri, diffusion, perfusion, spectroscopy

## Abstract

Purpose: Our aim was to determine the diagnostic performance of the combined usage of diffusion-weighted imaging (DWI), magnetic resonance spectroscopy (MRS) and perfusion MR (MRP) imaging for the differential diagnosis of benign and malignant intracranial lesions.

Materials and methods: A total of 30 patients with intracranial lesions who were prospectively evaluated with contrast-enhanced magnetic resonance imaging (MRI), DWI, MRS, and MRP were included in this study. The lesions were classified as benign and malignant according to the radiologic findings. All imaging data were compared with the histopathologic results and follow-up of the patients. We used the Pearson chi-square test and Fischer’s exact t-test for statistical analysis.

Results: For the differentiation of benign and malignant brain lesions, CBV and choline/creatine (Cho/Cr) ratio at short echo time (TE) had the highest sensitivity (87%-88%), Cho/N‐acetyl aspartate (NAA) at short TE had the highest specificity (86%). DWI predicted 77% sensitivity, 75% specificity; MRP presented 91% sensitivity, 88% specificity; MRS yielded 77% sensitivity, 63% specificity. The combination of either DWI and MRS, MRP and MRS or DWI+MRS+MRP revealed 100% sensitivity, 100% specificity.

Conclusion: For the differentiation of benign and malignant brain lesions, the combination of DWI, MRS, and MRP predicted 100% sensitivity. Invasive procedures like transcranial biopsy were not required via the usage of these advanced MRI techniques.

## Introduction

Non-invasive and accurate differentiation of brain mass lesions are important for determining the correct treatment plan and in some cases, may avoid the necessity of performing a biopsy [1-2]. Conventional magnetic resonance imaging (MRI) is a useful tool in evaluating the tumoral and non-tumoral brain lesions but not really sufficient for diagnosing all conditions [2].

Proton magnetic resonance spectroscopy (H-MRS), a non-invasive technique, has been used to observe metabolite changes in different intracranial abnormalities such as tumors, stroke, tuberculomas, multiple sclerosis and metabolic-inherited brain disorders, epilepsy and traumatic injuries [1-3]. Several types of non-neoplastic brain disorders (infectious-demyelinating lesions, etc.) can be potentially misdiagnosed as brain tumors. H-MRS may improve the diagnosis of unknown brain lesions [2-3]. Particularly H-MRS is added to the routine brain MRI in order to solve diagnostic problems such as differentiation of neoplastic and non-neoplastic lesions, low and high-grade tumors, ischemia from low-grade gliomas or discriminating the metastases from primary brain tumors and abscess [4-9].

Diffusion-weighted imaging (DWI), which is one of the functional MRI modality, allows quantitative measurement of the motion of water molecules in lesions and normal tissues. The net diffusion of the water molecules is referred to as the apparent diffusion coefficient (ADC). The high sensitivity and specificity of DWI in the diagnosis of acute cerebral infarction is widely accepted. Also, DWI has a diagnostic potential to evaluate brain tumors by using the ADC values [10-15].

Perfusion MRI (MRP) also aids conventional MRI in the diagnosis and follow-up of brain mass lesions. It is always problematic to judge whether a tumor is benign or malignant, primary or metastatic, and low or high grade. Despite some characteristic MRI findings, it can be difficult, sometimes even impossible, to distinguish between these tumors, and in such cases, gross tumor resection or stereotactic biopsy is required for precise diagnosis. MRP has long been used to overcome these problems in brain tumors [8,12,16-18]. It acquires quite important information for the classification of glial tumors, discrimination of recurrent tumors from radiation necrosis, and depiction of brain lymphomas via relative cerebral blood flow and volume (CBF-CBV) [6-9,16-18].

In this study, our aim was to determine the diagnostic performance of the combined usage of DWI, MRP, and MRS compared with conventional MRI for the differentiation of benign and malignant brain lesions.

## Materials and methods

Our research composed of 30 patients with intracranial mass lesions; 18 females and 12 males with age range 49-72, were analysed prospectively. DWI, H-MRS and MRP techniques were added to routine brain MRI in these patients. Local institutional ethics committee approved the research and a consent form was taken from all patients included in the study. The lesions were classified as benign and malignant according to the radiologic and histopathologic results. 

All the MRI, DWI-ADC mapping, MRS analysis, and MRP were carried out with an 8-channel 1.5 T MR scanner (Philips Achieva, Philips Medical systems, Netherlands) by using a standard head and neck array coil. Routine brain MRI, DWI, H-MRS and MRP techniques were performed one after each other, post-contrast series following pre-contrast sequences. In the routine brain MRI, T1‐weighted (T1W) axial, T2W axial and coronal, T2W Flair sagittal images were handled. Post-contrast T1W axial and coronal planes were added to routine imaging techniques.

For DWI-ADC mapping, axial plane echo-planar imaging (EPI) sequence with b factor=1000 s/mm² was applied and ADC mapping was performed with field of view (FOV) 23 cm, 256 x 77 matrix; all diffusion gradients were performed from x-y-z planes. ADC measurement was performed by manual implantation of region of interest (ROI) to the solid parts of mass lesions; three ROI measurements were done for each lesions, from anterior-paramedian and posterior sides then the average of them was considered as the final ADC values. For comparison and statistics, a measurement from contralateral normal white and gray matter were also handled; 5-7 mm² ROI sizes were used. Lesions with restricted diffusion and lower ADC values were considered to be malignant.

MRS was performed by using point-resolved spectroscopy (PRESS) with a volume of interest (VOI), 1 x 1 x 0.5 cm3 standard voxel sizes for the multivoxel spectroscopy and presaturation bands placed around the VOI. Depending upon the tumor and the lesion size, approximately 5-10 cm3 tumor area on the multivoxel imaging was harbored with the volume made up of such standard voxels. We have positioned the possible voxel within the solid tumoral or lesional area avoiding areas of cysts, normal appearing brain parenchyma, scalp or skull base contamination. Automatic shimming of the linear x, y, z channels was used to optimize field homogenity, water resonance and water suppression pulses were optimized for the consistent water saturation.

Proton spectrum was recorded in axial plane with repetition time (TR), 1500 ms; echo time (TE), 44 and 144 ms; FOV, 24 x 24 cm; 0.5 cm slice thickness; 256 x 256 matrix and 24 x 24 phase encoding. The duration of scan for both TE acquisitions was about five minutes. N-Acetyl aspartate (NAA) at 2 ppm, creatine (Cr) at 3-3.1 ppm, phosphocreatine (Cr2) at 3.8-3.9 ppm, choline (Cho) at 3.2 ppm, lipids (Lip) at 0.9-1.3 ppm, lactate (Lac) at 1.3-1.4 ppm, glutamate and glutamine (Glx) at 2.45 ppm, glycine and or myo-inositol (Gly-MI) at 3.6-3.75 ppm were analysed. NAA/Cr, Cho/NAA, Cho/Cr ratio quantifications were also performed. Contralateral reference voxel was placed just symetric to the center of the original brain lesion for the statistical comparison. High Cho metabolites, depressed NAA peak, high intralesional Cho/NAA and Cho/Cr ratio, and depressed NAA/Cr were considered as malignant [1-2].

For MRP, gadobutrol 0.1-0.2 mmol/kg with automatic enjection was used as the contrast agent. 100 ml isotonic SF was administered with dosage of 5 ml/sec. Six images were performed within 72 seconds after contrast enhancing. Axial plane EPI sequence was applied, post-processing of all images were acquired in the Philips work-station. 4-6 mm³ volumetric ROI’s were used intralesionally. CBV, CBF, the mean transit time of contrast agent (MTT) and time to peak (TTP) were expressed automatically by the system. Four ROI’s as an average were used for perfusion analysis of each mass lesions. A normal contralateral reference ROI was also applied for statistical correlation. Lesions with lower MTT and TTP, highly vascular with elevated CBV were diagnosed as malignant.

In the routine MRI, we expressed the enhancing pattern of lesions, heterogeneity-necrosis-perilesional edema and the nature of the lesions. All included sequences were interpreted by two neuroradiologists together with consensus. All statistical analyses were performed by using Statistical Package for the Social Sciences; version 15 (SPSS Inc., Chicago, IL).

Receiver operating characteristics (ROC) curve was also fitted to obtain cut-off values for ADC, Cho/Cr, NAA/Cr and NAA/Cho ratios to determine benign-malignant lesions by using area under this curve (AUC). Fischer’s exact t-test, Pearson chi-square test were used for statistical analysis of benign-malignant brain lesions. P<0.05 was considered to indicate significant statistical difference for all tests.

## Results

All eight malignant lesions (four high-grade glial tumor, two glioblastoma multiforme (GBM), a metastasis, and big cell lymphoma), and six of the benign lesions (two cavernomas and pilocytic astrocytomas, a central neurocytoma and hemangioblastoma) were verified by histopathology. The other benign lesions (four menengiomas and five ischemia, an arachnoid cyst, a radiation necrosis, an abscess-hematoma-encephalomalacia, and cystic extra-axial lesion) were correlated with radiologic and clinical follow-up. 

In the malignant group, ADC values were between 0.32-0.62×10-3 s/mm². Cho/NAA ratio was between 1.42-2.17 in short TE and 1.42-2.28 in long TE. Cho/Cr ratio was between 0.37-3.33 at short TE and 1.79-5.21 at long TE. NAA/Cr ratio was between 1.67-3.98 in long TE and 0.1-3.55 at short TE. CBV was between 627-831 ml, CBF between 37-71 ml/sec (Table 1).

**Table 1:** Cases and results in malignant lesions (Metabolite ratios in *: Long TE, **: Short TE values) (ADC: Apparent Diffusion Coefficient, CBV: Cerebral Blood Volume, CBF: Cerebral Blood Flow, NAA: N-Acetyl Aspartate, Cr: Creatine, Cho: Choline)

	High grade gliomas (6)	Metastasis (1)	Lymphoma (1)
ADC (×10‾³)	0.62	0.54	0.32
CBV	628	831	627
CBF	38	37	71
NAA/Cr*	3.55	0.1	1.75
Cho/Cr*	2.03	0.37	3.33
Cho/NAA*	1.56	1.42	2.17
NAA/Cr**	3.98	1.96	1.67
Cho/Cr**	5.21	1.79	2.8
Cho/NAA**	2.28	1.42	2.09

In the benign group, ADC values were between 0.36-3.21×10-3 s/mm², lowest in central neurocytoma-abscess and cavernoma, highest in encephalomalacia and cystic lesion. CBV was between 54-2297 ml, highest in hemangioblastoma, lowest in encephalomalacia. Cho/Cr, Cho/NAA ratios were highest in pilocytic astrocytomas and NAA/Cr ratio was high in most of the benign lesions (central neurocytoma, cavernoma, abscess, etc.) (Table 2).

**Table 2:** Cases and results in benign lesions (Metabolite ratios in *: Long TE, **: Short TE values) (ADC: Apparent Diffusion Coefficient, CBV: Cerebral Blood Volume, CBF: Cerebral Blood Flow, NAA: N-Acetyl Aspartate, Cr: Creatine, Cho: Choline)

	Pilocytic Astrocytoma (2)		Menengioma (4)		Hemangioblastoma (1)			Central neurocytoma (1)		Abscess (1)
ADC(×10‾³)	1.05		0.87		1.42			0.50		0.45
CBV	213		395		2297			360		70
CBF	7		32		252			38		13
NAA/Cr*	1.38		3.46		0.67			2.3		1.52
Cho/Cr*	3.05		1.93		1.56			0.59		0.41
Cho/NAA*	3.07		2.79		2.5			1.18		1.15
NAA/Cr**	1.66		2.1		1.23			2.76		1.37
Cho/Cr**	2.78		1.35		1.12			0.46		0.13
Cho/NAA**	2.48		0.97		1.22			0.33		0.73
	Cavernoma (2)	Hematoma (2)		Cystic lesion (2)		Ischemia (4)	Radiation necrosis (1)		Encephalomalacia (2)	
ADC(×10‾³)	0.36	1.06		2.68		1.0	1.13		3.21	
CBV	369	203		256		144	67		54	
CBF	32	15		18		13	8		10	
NAA/Cr*	4.8	3.11		2.74		2.6	2,17		1.96	
Cho/Cr*	1.63	0.98		1.39		1.9	0.67		0.74	
Cho/NAA*	0.79	0.95		0.85		0.6	0.55		0.36	
NAA/Cr**	3.52	3		1.7		2.53	2.03		1.79	
Cho/Cr**	1.03	0.48		0.93		1.24	0.45		0.6	
Cho/NAA**	0.77	0.23		0.68		1.11	0.73		0.78	

**Figure 1:**
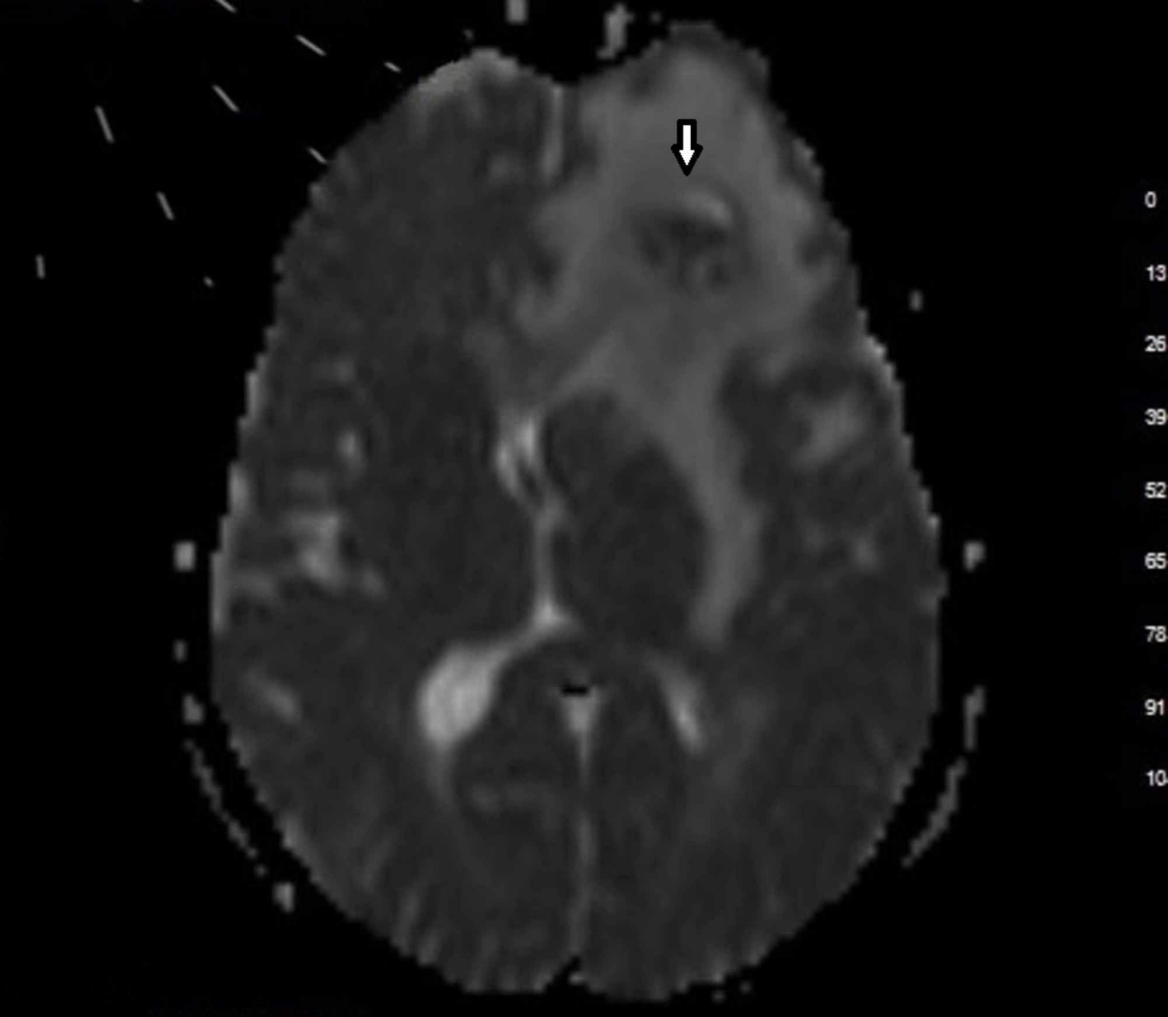
Restricted diffusion on diffusion-weighted imaging (white arrow) 65-year-old man with malignant epithelial tumor metastasis at the left frontal lob.

**Figure 2:**
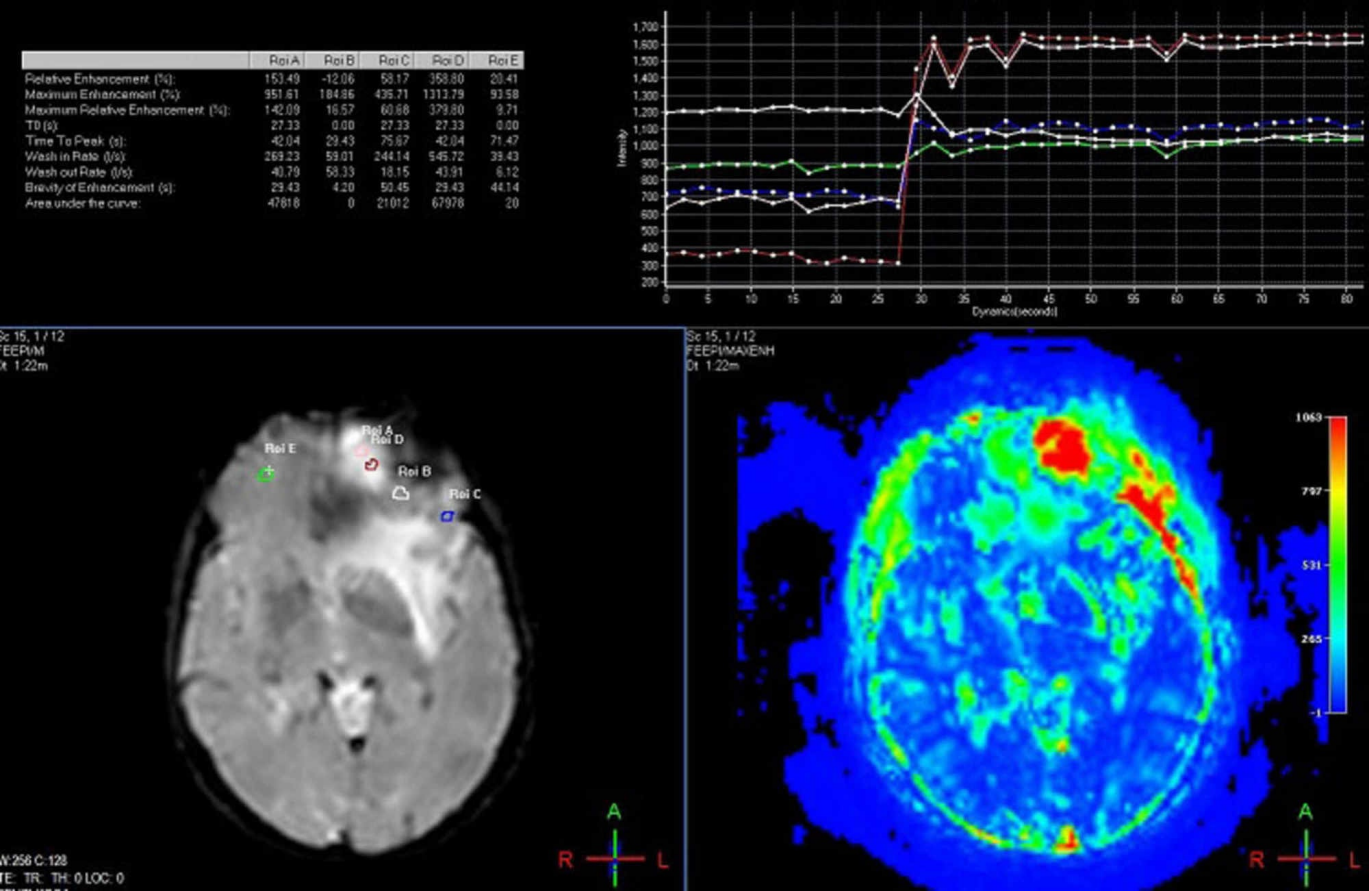
High vascularity on magnetic resonance perfusion (MRP) 65-year-old man with malignant epithelial tumor metastasis at the left frontal lob; ROI-A and ROI-D are intralesional and have high "maximum enhancement" ratios with low "time to peak" ratios, according to ROI-E (contralateral frontal lob), ROI-B and C (areas adjacent to the lesion). ROI: Region of Interest.

**Figure 3:**
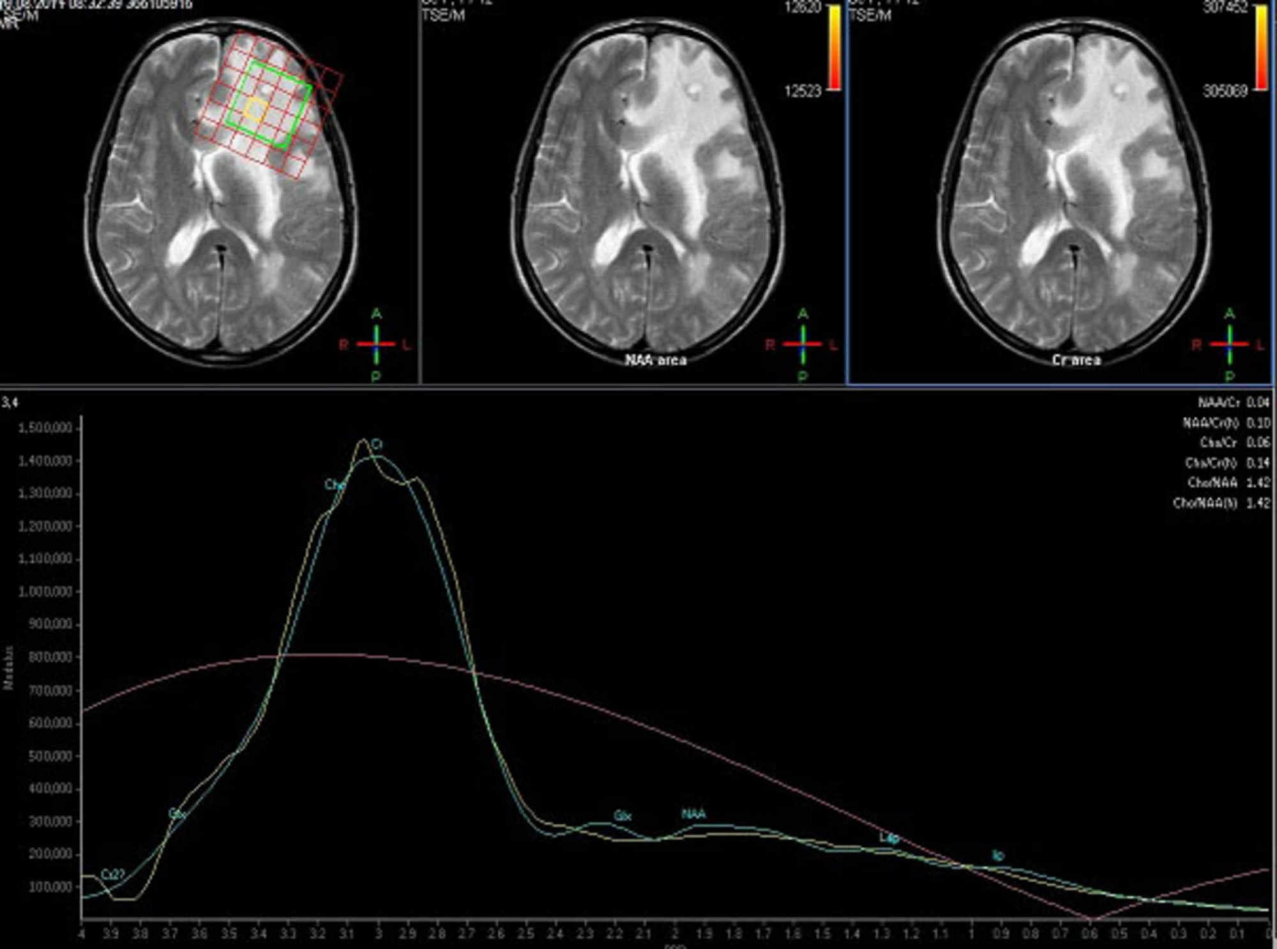
Dominant Cho-Cre metabolite peaks, low NAA and high Cho/NAA ratios on MRS as expected in malignant lesions 65-year-old man with malignant epithelial tumor metastasis at the left frontal lob. MRS: Magnetic Resonance Spectroscopy, NAA: N-Acetyl Aspartate, Cr: Creatine, Cho: Choline.

ADC values were significantly low in malignant lesions, CBV was quite high in malignant brain masses. Cho/Cr, Cho/NAA ratios were significantly high in the malignant group especially at long TE acquisition. NAA/Cr ratio was high in both malignant and benign groups at both short and long TE (Figures 1-3).

With the ROC curve analysis, cut-off values were determined: significant results were not obtained for ADC and NAA/Cr at long TE. Cut-off value was 406.50 for CBV, 29.50 for CBF; at long TE, 1.395 for Cho/NAA and 1.295 for Cho/Cr ratios; at short TE, 3.215 for NAA/Cr, 1.345 for Cho/NAA and 1.49 for Cho/Cr ratios. We had 87% sensitivity and 81% specificity for CBV, 75% sensitivity and 73% specificity for CBF, 62%-88% sensitivities and 68%-86% specificities for all ratios at both TE acqusitions (Table 3).

**Table 3:** DWI, MRP, MRS results via ROC curve analysis (Metabolite ratios in *: Long TE, **: Short TE values) DWI: Diffusio-weighted Imaging, MRP: Magnetic Resonance Perfusion, MRS: Magnetic Resonance Spectroscopy, ROC: Receiver Operating Characteristics, CBV: Cerebral Blood Volume, CBF: Cerebral Blood Flow, NAA: N-Acetyl Aspartate, Cr: Creatine, Cho: Choline.

	Cut-off value	Sensitivity (%)	Specificity (%)
CBV	≥ 406.50	87	81
CBF	≥ 29.50	75	73
Cho/Cr*	≥ 1.295	75	68
Cho/NAA*	≥ 1.395	75	77
NAA/Cr**	≤ 3.215	62	86
Cho/Cr**	≥ 1.49	88	77
Cho/NAA**	≥ 1.345	75	86

For the differentiation between benign and malignant lesions, CBV and Cho/Cr ratio at short TE had the highest sensitivites (87%-88%), Cho/NAA at short TE had the highest specificity (86%).

By conventional MRI, the lesions which were contrast-enhancing and had peripheral edema-mass effect were considered to be malign; 23 malignant and seven benign lesions were determined but unfortunately, only eight of them were proved to be malignant. All accepted benign lesions were true-positive; 32% sensitivity with 100% specificity was reported for routine MRI without any statistical difference to the exact diagnosis (p>0.05).

By ADC mapping and DWI, the lesions which showed restricted diffusion and lower ADC values ​​than normal brain parenchyma were considered to be malignant; the lesions which were isointense with cerebrospinal fluid (CSF), did not show restricted diffusion and had the same or higher ADC values ​​than normal brain parenchyma were considered to be benign. So that 11 malignant and 19 benign lesions were reported but only six of them were true positive malignant and two of the benign lesions were proved to be malignant. 77% sensitivity with 75% specificity for DWI-ADC mapping with significant statistical difference to the correct diagnosis, was revealed (p<0.05).

By MRP, the measurements made from the lesions, MTT, TTP and CBV values were compared with the measurements of non-pathological brain parenchyma. The lesions showing increased vascularization and high CBV values ​​were considered to be malignant. Nine of 30 lesions were expressed as malignant and 21 of 30 lesions were considered as benign. Seven of them were true-positive malignant and one of the considered benign lesions was false-positive, verified as malignant. MRP had 91% sensitivity and 88% specificity with a significant statistical difference to the correct histopathologic diagnosis (p<0.05). 

By MRS, the measurements from the lesions made in long and short TE values were compared with the measurements of non-pathological brain parenchyma. According to the metabolite ratios and predominant metabolite peak, the lesions were divided into benign and malignant. The lesions without increased Cho, without NAA depression and with the predominant metabolite NAA were considered to be benign. The lesions with NAA depression, with increased Cho/Cr and Cho/NAA ratios, with the predominant metabolite Cho were considered to be malignant. So that 10 lesions were predicted as malignant and 20 lesions were determined as benign but only five of the 10 lesions were true-positive malignant and three of the benign lesions were verified as malignant. MRS had 77% sensitivity and 63% specificity without any statistical difference to the exact histopathology (p>0.05).

 By the combination of DWI and MRP, 23 lesions were classified as benign and seven lesions were determined as malignant, all malignant lesions were true-positive malignant but only one of the benign considered lesion was proved to be malignant. DWI+MRP revealed 100% sensitivity and 88% specificity with significant statistical differences to the exact histopathology and biopsy (p<0.05).

As a whole, conventional MRI and DWI had the lowest sensitivities and specificities. By the combination of either DWI and MRS, MRP and MRS or DWI+MRS+MRP revealed 100% sensitivity and 100% specificity.

## Discussion

MR imaging is the standart technique for brain tumor diagnosis. Conventional MRI parameters such as enhancement, mass effect, and signal intensity heterogeneity were correlated with malignancy, but not always effective. Advanced MR techniques (DWI, MRP, and MRS) called functional, metabolic, hemodynamic and cellular methods are used in the assessment of the brain lesions. We can use them in grading the neoplastic lesions and the determination of the treatment.

DWI has been applied for the assignment of tumor grades or differentiation of tumors, as well as for the diagnosis of ischemic stroke [5,10-16,19]. Several investigators found an inverse correlation between the ADC calculated from DW images and tumor cellularity [5,13-14,16]. Lower ADC values were accepted as a marker of higher tumor grades [13-15,18]. In our experience, ADC values were lower in malignant tumors than benign tumors [8,12,14,18]. The high-grade gliomas and the metastases had similar ADC values (0.62×10‾³ and 0.54×10‾³); it was higher in menengiomas (0.87×10‾³) and the highest in the pilocytic astrocytoma (1.05×10‾³); lower in abscess (0.45×10‾³) and the lowest in lymphoma (0.32×10‾³) [5,13, 15]. The abscess should be considered as malignant according to its ADC value, but low CBV value, high NAA/Cr and low Cho/Cr and Cho/NAA ratios showed that it was a benign lesion.

MRS has the non-invasive potential to provide additional metabolic information to improve pre-surgical diagnosis [2]. MRS can also help to identify the tumor type and grade. The presence of the lactate and lipid peaks were usually consistent with aggressive tumors, reflecting increased anaerobic metabolism and cellular necrosis, respectively [5,8-9]. We found that in the distinction of the brain lesions as benign or malignant the Cho/Cr ratio (cut-off value 1.49) calculated in short TE values had the highest sensitivity (88%), while the specificity was 77%. The Cho/NAA ratio (cut-off value 1.345) calculated in short TE values had the highest specificity (86%), while the sensitivity was 75%. The Cho/Cr (3.773) and Cho/NAA (1.87) ratios were higher in the malignant group than in the benign group (1.064; 0.84). These results are similar to the literatüre [4,6-7,20-21]. However, the increased NAA/Cr ratios (3.13) in our malignant group (2.20 in the benign group) are not consistent with the literature; this may be because of the necrotic areas and the heterogeneity of high-grade gliomas in our study. The NAA/Cr ratio had lower sensitivity in the distinction of the lesions as benign or malignant (62%).

The third MRI modality we used in our study was MRP which provides noninvasive physiological measurements of tumor vascularity and relative CBV maps that can be used to identify areas of neovascularization [6,9,12,17,22]. For this reason, high-grade neoplasms, which are associated with a high degree of neovascularity, have been shown to have high capillary density and, on that basis, high rCBV [7,12,17-18]. MRP may provide an objective measurement for distinguishing radiation necrosis from recurrence after treatment of cerebral neoplasms [17]. In radiation necrosis, very low CBV values are detected ​​due to the vascular damage, while high CBV values ​​are detected in tumors. In our study, the highest CBV value was detected in hemangioblastoma (2297 ml) and respectively metastasis (831 ml), high-grade gliomas (628 ml) and lymphoma (627 ml); the lowest value was detected in pilocytic astrocyitoma (213 ml). In distinction of the lesions as benign or malignant, the sensitivity and specificity values for CBV (cut-off value 406,5) were 87%-81% and for CBF (cut-off value 29,50) were 75%-73%.

In the literature, some authors reported that the combination of DWI (ADCmin) and MRP (rCBVmax) could be more effective in the differential diagnosis of cerebral tumors [12,18]. The combination of ADC values and MRS findings could be more useful in staging and differential diagnosis of cerebral tumors [5,19]. Additionally, the combination of rCBV and metabolite ratios should be used in differentiating neoplastic-nonneoplastic brain lesions and in grading cerebral gliomas [3,6-7,9]. It had increased the sensitivity compared with conventional MRI.

The present study found out that in differentiating neoplastic-nonneoplastic brain lesions the combination of DWI, MRS and MRP was more benefical than using them alone. DWI predicted 77% sensitivity and 75% specificity, MRP presented a 91% sensitivity and 88% specificity, MRS yielded 77% sensitivity and 63% specificity; while the combination of DWI and MRP predicted 100% sensitivity and 88% specificity. By the combination of either DWI and MRS, MRP and MRS or DWI+MRS+MRP revealed 100% sensitivity and 100% specificity.

Limitations

All brain mass lesions were analysed by two radiologists together with consensus, without any intra and interobserver variability which might affect the statistical research, resulting variabilities in the sensitivity and specificity. 14/30 mass lesions were proved by histopathology, remaining 16 were correlated and verified by clinical follow-up and radiologic imaging. This might also cause changes in the sensitivity and specificity of DWI-MRS and MRP.

Small number of cases (30) might also affect the statistical research; results should have to be validated in other studies with a larger number of series.

## Conclusions

The usage of just conventional MRI is not enough for the differential diagnosis of intracranial mass lesions. High MR imaging techniques such as DWI, MRS, MRP were superior than conventional MRI and a combination of these sequences predicted 100% sensitivity for the differentiation of benign and malignant lesions. Without intravenous contrast agent administration, DWI+MRS regarded 100% sensitivity for the diagnosis of benign and malignant mass lesions. DWI+MRP, MRP+MRS combinations also revealed 100% sensitivities for the discrimination of benign and malignant mass lesions so invasive procedures like transcranial biopsy were not required via the usage of these advanced MRI techniques.
